# Patient-derived DIPG cells preserve stem-like characteristics and generate orthotopic tumors

**DOI:** 10.18632/oncotarget.19656

**Published:** 2017-07-28

**Authors:** Cheng Xu, Xiaoqing Liu, Yibo Geng, Qingran Bai, Changcun Pan, Yu Sun, Xin Chen, Hai Yu, Yuliang Wu, Peng Zhang, Wenhao Wu, Yu Wang, Zhen Wu, Junting Zhang, Zhaohui Wang, Rui Yang, Jenna Lewis, Darell Bigner, Fangping Zhao, Yiping He, Hai Yan, Qin Shen, Liwei Zhang

**Affiliations:** ^1^ Department of Neurosurgery, Beijing Tiantan Hospital, Capital Medical University, Beijing, China; ^2^ Center for Life Sciences, Center for Stem Cell Biology and Regenerative Medicine, School of Medicine, Tsinghua University, Beijing, China; ^3^ Department of Pathology, Duke University Medical Center, The Preston Robert Tisch Brain Tumor Center, The Pediatric Brain Tumor Foundation Institute, Durham, North Carolina, USA; ^4^ Peking-Tsinghua-NIBS Graduate Program, School of Life Sciences, Tsinghua University, Beijing, China; ^5^ Genotron Health (Beijing) Co. Ltd, Beijing, China

**Keywords:** DIPG, pre-clinical model, patient-derived cell line, orthotopic xenograft, pediatric brain tumor

## Abstract

Diffuse intrinsic pontine glioma (DIPG) is a devastating brain tumor, with a median survival of less than one year. Due to enormous difficulties in the acquisition of DIPG specimens and the sophisticated technique required to perform brainstem orthotopic injection, only a handful of DIPG pre-clinical models are available. In this study, we successfully established eight patient-derived DIPG cell lines, mostly derived from treatment-naïve surgery or biopsy specimens. These patient-derived cell lines can be stably passaged in serum-free neural stem cell media and displayed distinct morphologies, growth rates and chromosome abnormalities. In addition, these cells retained genomic hallmarks identical to original human DIPG tumors. Notably, expression of several neural stem cell lineage markers was observed in DIPG cell lines. Moreover, three out of eight cell lines can form orthotopic tumors in mouse brainstem by stereotactic injection and these tumors faithfully represented the characteristics of human DIPG by magnetic resonance imaging (MRI) and histopathological staining. Taken together, we established DIPG pre-clinical models resembling human DIPG and they provided a valuable resource for future biological and therapeutic studies.

## INTRODUCTION

Diffuse intrinsic pontine glioma (DIPG) affects approximately 200-300 children in the U.S. each year. The majority of patients are diagnosed between five and ten years old, with a median survival of less than one year [1]. The standard care for newly diagnosed DIPG patients is fractionated focal intensity modulated radiation therapy (IMRT) which only provides temporary relief of neurological symptoms and has little improvement on overall survival. Hyper-fractionated RT and hypo-fractionated RT have been used by several groups, but neither provides survival advantage. The use of RT concurrently with radio-sensitizers has shown a similar result. Numerous other treatment methods have failed to show survival improvement, including chemotherapy [2], concurrent chemoradiotherapy using temozolomide [3, 4], and several targeted therapies employed in clinical trials [5, 6]. The scarcity of DIPG pre-clinical models is perhaps the main reason for this lack of therapeutic progress.

Tumor models play a vital role in discovering more effective DIPG treatment methods. They provide us with insights into the biological characteristics of glioma and enable us to test novel therapeutic strategies. Given the genetic and micro-environmental distinctions between DIPG and supratentorial glioma, establishing a specific DIPG pre-clinical model is critical. However, due to scarcity of human DIPG specimens and the sophisticated technique required to perform brainstem orthotopic injection, the development of pre-clinical DIPG models has fallen far behind that of supratentorial glioma models.

DIPG pre-clinical models began with allograft models generated from murine glioma cell lines, such as F98 and C6, resulted in the finding that tumors could be formed within the brainstem [7–11]. Human glioblastoma xenograft models were later developed by using human adult cortex glioma cell lines, giving rise to tumors that resembled some characteristics of human DIPG [12–14]. These two types of models suggested the feasibility of brainstem injection and took into consideration the specific brainstem microenvironment. However, generated by cortex glioma cells, either from mouse or human, these models could not be regarded as appropriate systems to test DIPG therapeutic strategies due to the significant biological distinction between cortex and brainstem gliomas.

Monje et al. developed patient-derived DIPG cell lines and orthotopic xenografts using autopsy specimens [15]. Interestingly, they found that direct injection of human DIPG cells without a prior culture gave rise to murine tumors, while indirect injection after *in vitro* culture gave rise to tumors consisting of human cells [16]. One caveat of these models was that a DIPG autopsy specimen was usually previously exposed to radiotherapy and other treatments, leading to genetic shifts in the tumor and possibly affecting the reliability of using these models for drug screening. To circumvent this dilemma, cell lines derived from DIPG biopsies have been established [17–20]. Notably, Hashizume et al. modified patient-derived DIPG cells with hTERT and a luciferase reporter and generated brainstem xenograft models resembling the genomic features of human DIPG [21]. Several *in vitro* and/or *in vivo* tests for DIPG-targeted therapies were conducted based on these models [20, 22, 23].

Other than patient-derived models, DIPG genetically engineered mouse models (GEMMs) using a replication-competent avian sarcoma-leucosis virus long-terminal repeat with splice acceptor (RCAS)/tumor virus A (TVA) modeling system was also reported [24]. Funato et al. used a human embryonic stem cell system with H3.3K27M expression, p53 loss and PDGFRA activation to model DIPG both *in vitro* and *in vivo* [25]. GEMMs and human embryonic stem cell systems were important tools to study the function of DIPG driver mutations, but the weakness was that they cannot faithfully represent complete genetic features of DIPG tumors.

Despite several groups showed the possibility of DIPG autopsy and the feasibility of DIPG biopsy aiming to acquire sufficient specimens for conducting further research, DIPG pre-clinical resources are still extremely limited compared to supratentorial ones, especially the cohort of patient-derived cell lines and xenograft models following the same protocol. In this study, we established eight DIPG cell lines from treatment-naïve specimens. These cells demonstrated variations in morphology, proliferation capacity and chromosome abnormality. Importantly, these cells retained gene mutations from original DIPG tumors and expressed several neural stem cell markers. With these patient-derived cell lines, brainstem orthotopic xenografts were successfully established and their imaging and pathological features were confirmed by MRIs and histopathological staining.

## RESULTS

### Clinical information

**Figure 1:**
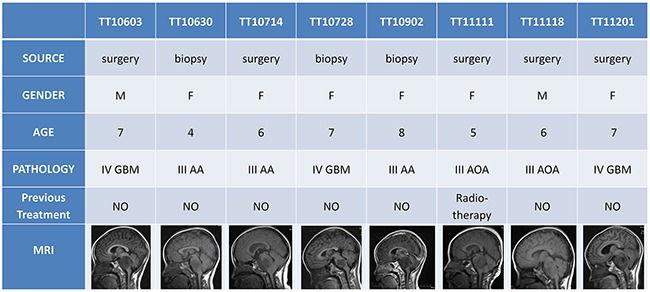
Clinical information of the patients Most of the DIPG patients were treatment naïve (except TT11111) when surgery or biopsy were performed. Histopathology showed that all patients were diagnosed as high-grade gliomas (3 cases of grade III anaplastic astrocytoma AA, 2 cases of grade III anaplastic oligodendroastrocytoma AOA, and 3 cases of grade IV glioblastoma GBM). MRI revealed the infiltrative tumors in pons and the invasion to midbrain, medulla oblongata, and cerebellum.

Tumor tissues were obtained from eight DIPG patients. The average age of these patients at diagnosis was 6.25 years old. Two patients were male and the other six were female. Five out of eight tumor tissues were obtained from surgery and the other three were from MRI-guided stereotactic biopsy. The histopathologic diagnoses of these tumor samples were singular (Anaplastic astrocytoma 3/8, anaplastic oligodendroastrocytoma 2/8, and glioblastoma 3/8), but all of them were high-grade gliomas (WHO III and WHO IV). Except for TT11111, who previously received radiotherapy, all other patients were treatment-naïve. The MRI scans of these patients demonstrated infiltrative tumors in pons and the invasion to midbrain, medulla oblongata, and cerebellum (Figure 1).

### Establishment of DIPG cell lines and characterization of cell morphology

**Figure 2:**
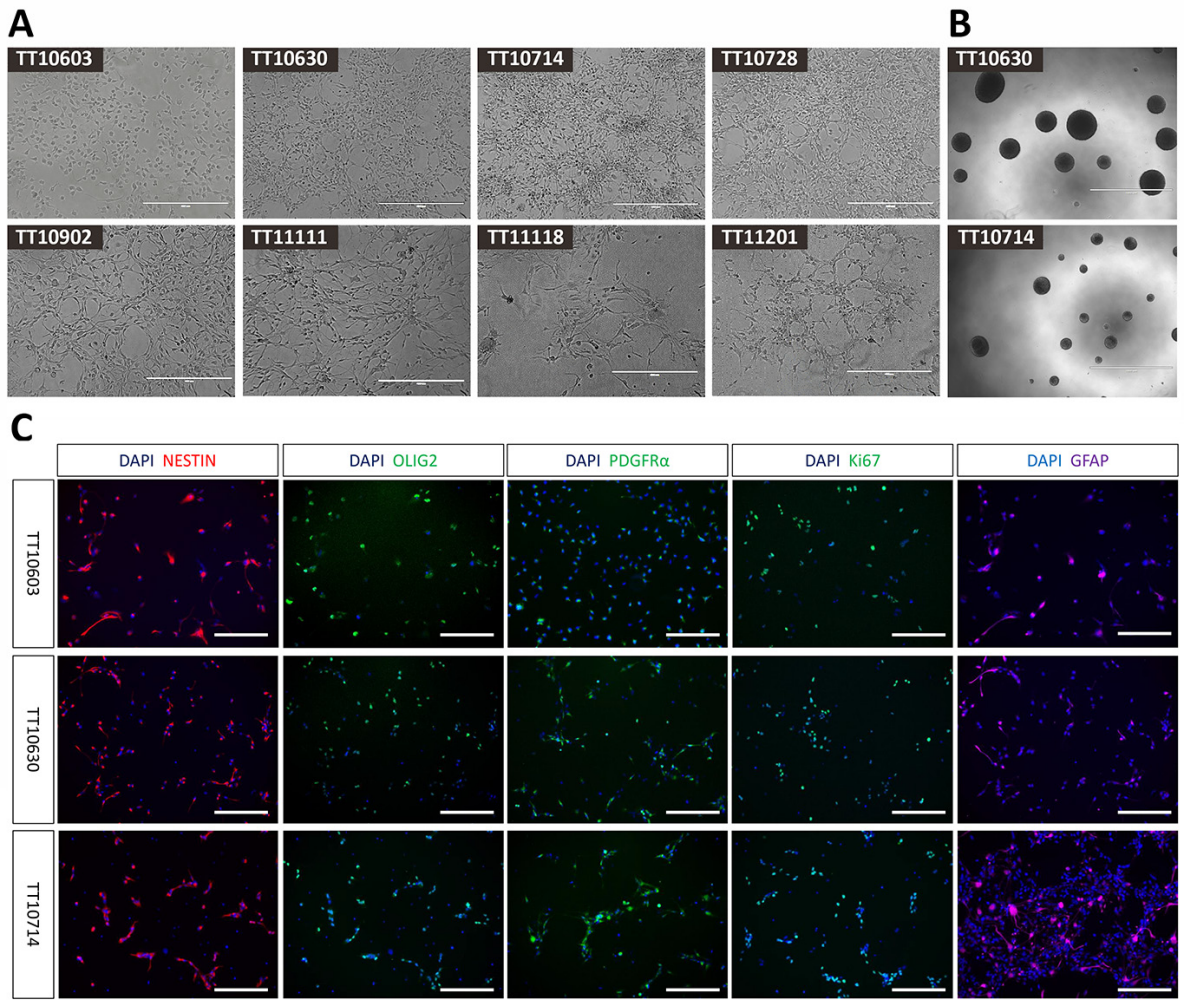
The morphology and immunophenotype of DIPG cell lines **(A)** Cell lines derived from different DIPG samples displayed distinct morphologies (20X), including flattened or round, bipolar with long processes and multipolar shapes. (Scale bars: 400μm). **(B)** Neurosphere formations of representative DIPG cell lines (4X). (Scale bars: 1000μm). **(C)** Immunocytochemistry staining of representative DIPG cell lines. Positive staining of neural stem cell marker (Nestin), oligodendrocyte progenitor cell markers (Olig2 and PDGFRα) and astrocyte marker (GFAP) demonstrated neural progenitor status of established DIPG cells (20X). The positive staining of proliferation marker Ki-67 confirmed the proliferative property of these cells. (Scale bars: 200μm).

DIPG tissues were obtained following surgical or biopsy procedures in Beijing Tiantan Hospital and were immediately processed (see Materials and Methods). The protocol was approved by the human research ethics committee of Beijing Tiantan Hospital and written informed consent was obtained from the subjects’ parents. After the digestion process, dissociated single-cell suspensions were cultured in Poly-L-ornithine (PLO)/Laminin-coated plates with serum-free neural stem cell medium. We observed that only a sub-population of cells were able to survive and proliferate after initial plating and these cells formed tight small clusters of cells at the first passage. These small aggregates then spread to become more flattened clones. After three to five passages, these cells were free of cell debris and showing as monolayer cells with homogenous morphology and division rate for each cell line. In total, 25 samples were harvested, digested, and cultured and 8 samples could be expanded for more than 20 passages, maintaining stable growth rates and morphology. In these 25 samples, 14 of them were high grade gliomas and 11 of them were low grade gliomas. The success rate of high grade group (8/14) was significantly higher than that of low grade group (0/11). This result indicated that tumor grade contributed the most in determining whether brainstem glioma cell lines can be established successfully. We also observed efficient recovery of each cell line following freezing and thawing. Cell lines derived from different tumor samples displayed different morphologies, indicative of heterogeneity of original brainstem gliomas. These diverse cell morphologies can be roughly categorized as: (1) flattened and/or round, (2) bipolar with long processes, or (3) multipolar (Figure 2A). Moreover, to test the neurosphere forming capacity, 1,000 cells were plated into 96-well plates without PLO/Laminin coating and cultured with the same serum-free media. After 14 days, all eight cell lines were capable of forming neurospheres, indicating their neural precursor status (Figure 2B).

### Immunocytochemistry staining of DIPG cell lines

To determine whether the glioma-derived cells had progenitor cell-like characteristics, we analyzed the expression of several neural stem cell lineage markers on these DIPG cells. By immunocytochemistry we found that most of the cells were positive for Nestin, a neural stem cell marker [26]. Interestingly, the oligodendrocyte progenitor cell markers Olig2 and PDGFRα, as well as GFAP, an astrocyte marker, were also highly expressed in cultured cells. The majority of cells were proliferating as revealed by positive Ki67 staining (Figure 2C, Supplementary Figure 1). The percentages of positive cells of Nestin, Olig2, PDGFRα, GFAP and Ki-67 were 59.2%-97.8%, 40.1%-95.0%, 36.3%-89.3%, 39.4%-82.6% and 36.6%-84.3%, respectively (Table 1). The result of single factor analysis of variance (ANOVA) demonstrated significant differences (*P<0.05*) between 8 DIPG cell lines in each marker. Thus, DIPG cells we established showed broad molecular similarities to normal neural progenitor cells (NPCs) or oligodendrocyte precursor cells (OPCs).

**Table 1:** Summary of the characteristics of DIPG cell lines, including gene mutation, doubling time, choromosome abnormality, immunophynotype and tumorigenecity

	H3F3A	TP53	PPM1D	Doubling time(hours)	Chromosome structural abnormality	Chromosome numerical disorder	Nestin(%)	Olig2(%)	PDFGRα(%)	GFAP(%)	Ki-67(%)	Orthotopic xenograft
TT10603	K27M	R141C	-	67.6±4.2	-	1,2,3,4,6,7,9,10,12,13,15,16,17,1820,21,22,X	76.2±6.5	43.8±1.3	82.3±1.1	45.4±6.9	61.4±5.7	3/3
TT10630	K27M	-	S516X	43.1±1.6	15	-	71.3±3.5	57.9±1.8	75.7±15.4	39.4±3.1	84.3±5.7	3/3
TT10714	K27M	-	C478X	37.1±0.3	2,7,8,9,13,14	-	72.6±4.1	40.1±0.7	79.4±1.3	43.9±0.7	49.7±1.7	3/3
TT10728	K27M	R116W	-	46.8±0.4	-	4,6,11,13,14,15,21,22	89.3±1.2	70.2±1.1	71.3±0.8	82.6±9.0	36.6±4.2	0/3
TT10902	K27M	R141C	-	54.9±0.1	-	1,2,3,5,7,8,9,10,11,12,13,14,15,18,19,20,X	68.0±1.0	95.0±1.0	89.3±1.3	74.3±16.6	53.1±1.0	0/3
TT11111	K27M	-	-	49.0±0.8	-	3,10,12,13,1618,21,22	97.8±0.4	91.1±0.7	36.3±2.9	81.1±0.8	36.6±2.9	0/3
TT11118	K27M	V25F	-	70.9±4.7	1,4	3,7,18,19,22	59.1±4.5	90.1±1.0	45.7±2.5	64.2±1.0	74.1±2.1	0/3
TT11201	K27M	Y31C	-	49.5±0.2	-	1,2,3,4,6,9,10,11,13,14,15,16,17,18,20,21,22,X	97.0±1.0	74.1±1.0	53.9±1.9	46.8±1.9	71.9±0.4	0/3

### Gene mutation status in DIPG cell lines and original tumor tissues

**Figure 3:**
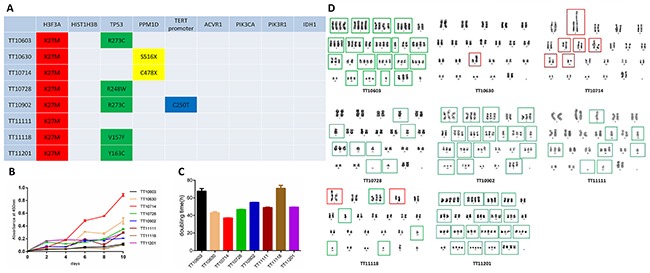
The genomic signature, proliferation capacity and chromosome abnormality of DIPG cell lines **(A)** Sanger sequencing demonstrated thatestablished DIPG cell lines retained gene mutations in *H3F3A*, *TP53* and *PPM1D* which were consistent with their matching human DIPG tumors. **(B-C)** Proliferation capacities of DIPG cell lines were measured by growth curves and doubling times. **(D)** Karyotype analysis of established DIPG cell lines. TT10630 and TT10714 manifested as significant structural abnormalities (red frames) with normal overall chromosome quantity, while TT10603, TT10728, TT10902, TT11111 and TT11201 represented severe numerical disorders such as haploid and polyploid (green frames). TT11118 demonstrated both structural and numerical abnormalities.

To compare the mutation profile of established cell lines with their original tumor tissues, Sanger sequencing was performed to analyze the status of common mutations reported for DIPGs. Sequencing results showed that all eight cell lines harbored *H3F3A*-K27M mutation, the most frequent mutation identified in DIPGs. In addition, we identified five cell lines with *TP53* mutations (TT10603, TT10728, TT10902, TT11118, and TT11201), two cell line with *PPM1D* mutation (TT10630 and TT10714) and one cell line with wild type for both *PPM1D* and *TP53* (TT11111). Consistent with a previous study [27], *PPM1D* and *TP53* mutations were mutually exclusive. Interestingly, one line harbored a *TERT* promoter mutation. No mutation in *HIST1H3B*, *ACVR1*, *PIK3CA*, *PIK3R1* and *IDH1* was discovered in eight cell lines. Most importantly, all genetic status in cell lines matched their original tumor tissues (Figure 3A).

### Growth rate of DIPG cell lines

The growth rates of the DIPG cell lines were assessed by the CCK-8 assay. Almost all of the cell lines grew slowly during the initial four days, and the growth rate increased significantly afterwards. The doubling time of each cell line ranged from 26-60h (Figure 3B and 3C). Among the 8 cell lines, we noticed that TT10714 and TT10630 cells grew significantly faster than others. Interestingly, despite a long doubling time (second longest among 8 cell lines), TT01603 still could form intracranial tumor once being injected into mice. This discrepancy between *in vitro* growth rate and *in vivo* tumorigenesis capacity was consistent with a previous report [28]. The result of single factor ANOVA demonstrated statistically significant differences (P<0.05) in doubling times of 8 DIPG cell lines.

### Karyotype analysis of DIPG cell lines

Distinct chromosome abnormalities between different cell lines were observed by karyotype analysis. TT10630 and TT10714 manifested as significant structural abnormalities with normal overall chromosome quantity, while TT10603, TT10728, TT10902, TT11111 and TT11201 represented severe numerical disorders such as haploid and polyploid. TT11118 demonstrated both structural and numerical abnormalities (Figure 3D).

### Orthotopic xenografts can be generated by DIPG cell lines

**Figure 4:**
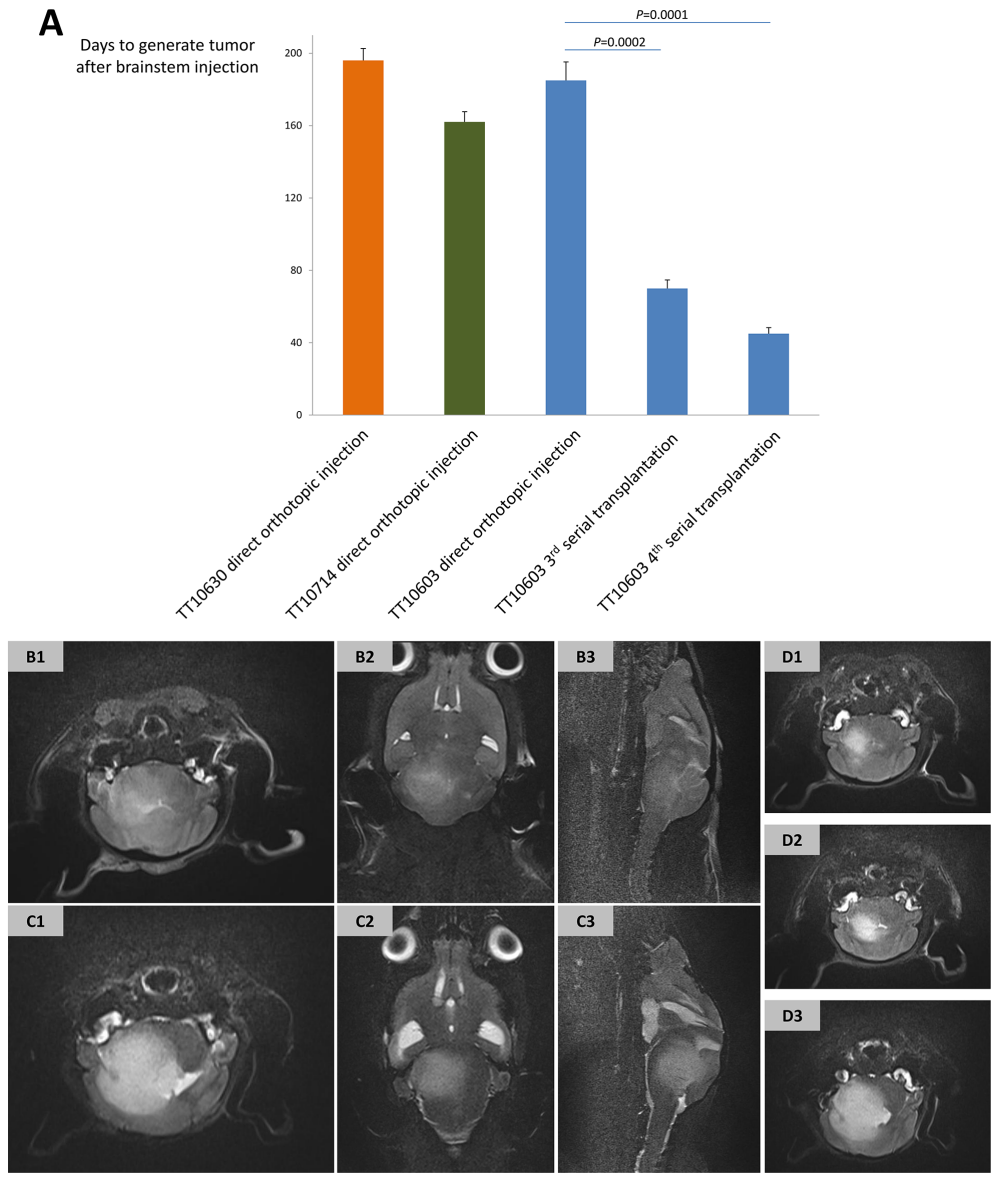
The establishment of DIPG xenograft models and their MRI manifestations **(A)** The latency of forming DIPG xenografts by direct orthotopic injection of TT10603, TT10630 and TT10714 cells was 172, 186 and 155 days post injection, respectively. Third and fourth generation of serial transplantations of TT10603 cells after subcutaneous propagation showed a significant shorter latency of 67 and 40 days. **(B-D)** Representative MRI of orthotopic xenograft acquired from 7.0-T mouse MR machine. **(B)** T2-weighted sequence of orthotopic xenograft generated by direct injection of TT10603 cells (B1: axial; B2: coronal; B3: sagittal). **(C)** T2-weighted sequence of serial xenograft generated by subcutaneously propagated TT10603 cells (B1: axial; B2: coronal; B3: sagittal). **(D)** Axial T2-weighted sequence of TT10603 orthotopic xenografts with different cell numbers (D1: 1,000 cells. D2: 10,000 cells. D3: 100,000 cells) on the 70^th^ day after injection.

To assess the capacity of orthotopic xenograft formation, DIPG cells were injected into brainstem area of immunocompromised mice (n=3 for each cell lines). Mouse MRI was performed on a 7.0-T machine with axial, sagittal and coronal T2-weighted sequences every 2 weeks or whenever a symptom was found. Three out of eight cell lines (TT10603, TT10630 and TT10714) formed tumors within the brainstem area, on 172, 186, and 155 average days after injection, respectively (Figure 4A). MRIs demonstrated large mass located in the pontine area with infiltration into the midbrain, medulla oblongata, and cerebellum (Figure 4B). Symptoms like hemiparalysis and weight loss were found in the mice suffering from tumors.

### Serial transplantation of the subcutaneously propagated cells had significantly short latency

Since the latency of forming a tumor with *in vitro* cultured cells was relatively long, we took another strategy by propagating cells in subcutaneous xenografts prior to orthotopic injection. While all eight cell lines were injected into the flanks of immunocompromised mice, only TT10603 and TT10630 cells generated subcutaneous tumors. The TT10603 subcutaneous tumor was then digested and injected into the mouse brainstem. Interestingly, the mice received serial transplantation displayed symptoms and MRI characteristics much earlier than the mice with direct orthotopic injection. The third and fourth serial transplantation of the TT10603 cell line took only 67 and 40 average days, respectively, to generate orthotopic tumors, representing a significantly decreased latency compared to direct orthotopic injection (Figure 4A). The MRI showed that while serial transplantation displayed similar features compared to the direct orthotopic injection group, it manifested a more intensive signal on the T2 sequence and invaded to broader region in the brain (Figure 4C).

### A limited quantity of DIPG cell was sufficient to initiate orthotopic tumor formation

To further test the stem-cell like characteristic of DIPG cell lines, TT10603 cells at different numbers (10^3^, 10^4^, and 10^5^) were injected into brainstem after subcutaneous propagation. Mice in all the groups formed tumors within 70 days, verifying that orthotopic xenografts can be initiated even by a small amount of DIPG cells (Figure 4D).

### Histopathological characteristic of mouse xenograft matched original human DIPG tissue

**Figure 5:**
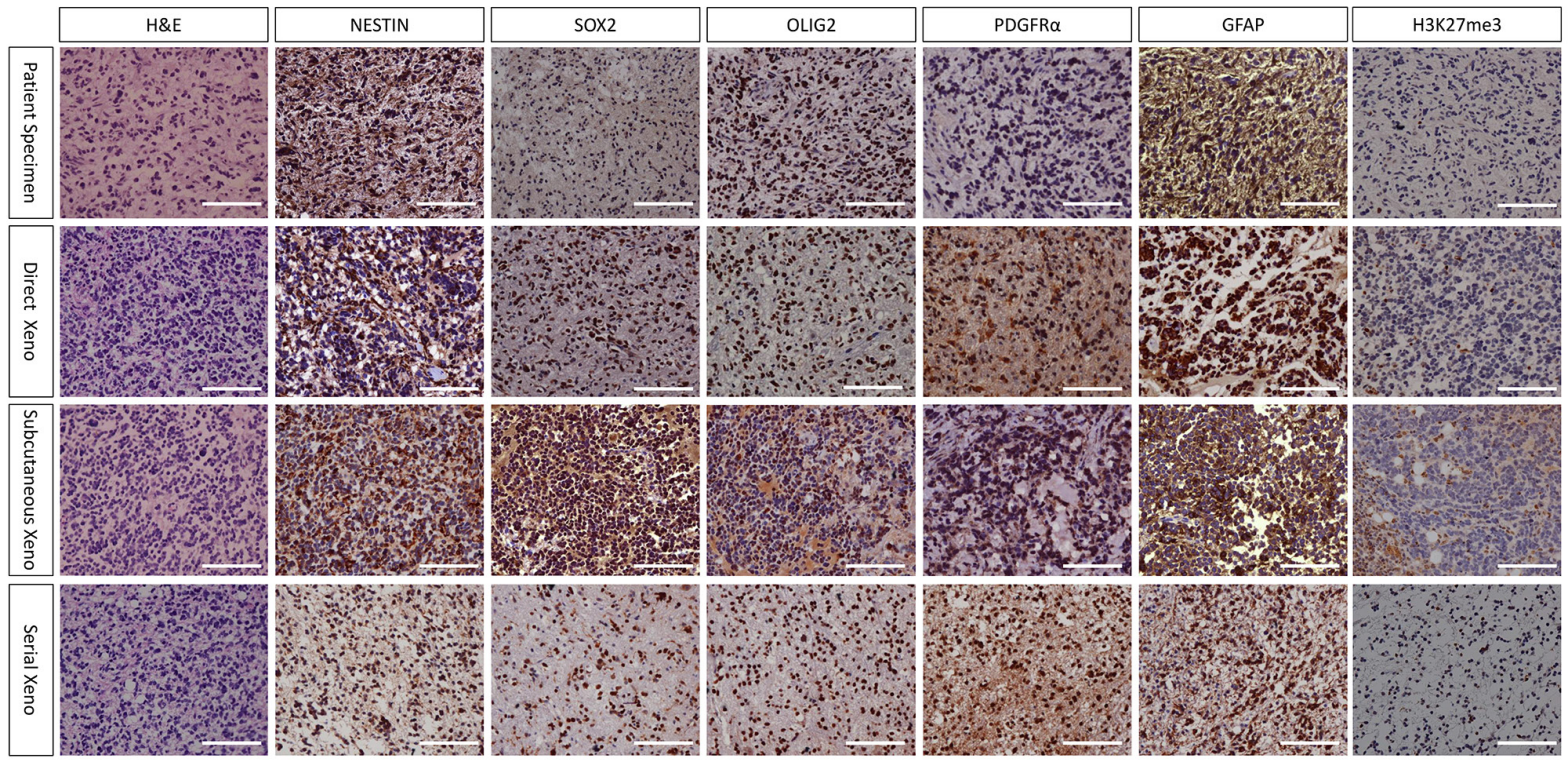
Histopathological characteristic of TT10603 patient specimen and mouse xenograft (20X) H&E staining of all xenografts revealed high cell density similar to human DIPG tissues, representing the characteristic of high grade glioma. Immunohistochemistry showed that all xenografts as well as their human DIPG counterparts were positive for Nestin, Sox2, Olig2, PDGFRα and GFAP. Notably, serial xenograft demonstrated higher percent of immunoreactivity for these markers than direct orthotopic xenograft. Most tumor cells of xenografts were negative in H3K27me3 staining, indicating that they retained the loss of trimethylation at position 27 of histone H3 from human tumor tissues. (Scale bars: 200μm).

The histopathological features of xenografts from direct orthotopic injection, subcutaneous injection and serial transplantation and their corresponding patient DIPG tissue were compared by H&E and immunohistochemistry staining. H&E staining of mouse xenografts revealed a high cell density similar to human DIPG tissues, representing the characteristic of high-grade glioma. Immunohistochemistry showed that both mouse orthotopic xenografts and their human DIPG counterparts were positive for neural stem cell markers including Nestin, Sox2, Olig2, PDGFRα, and GFAP (Figure 5, Supplementary Figure 2). Notably, xenografts from serial transplantation demonstrated nearly one hundred percent immunoreactivity for these stem cell markers, much higher than direct orthotopic xenograft. The expression of these markers was also observed in cultured DIPG cell lines (Figure 2C).

### Orthotopic xenografts retained *H3F3A* mutation and hypomethylated K27 residue

Xenograft tissue was harvested for DNA extraction and Sanger sequencing was performed to analyze the status of *H3F3A* mutations. All xenografts from direct orthotopic injection (TT10603, TT10630 and TT10714) and xenografts from serial transplantations (TT10603) retained the *H3F3A*-K27M mutation of their original tumors. Immunohistochemistry staining of H3K27me3 demonstrated that orthotopic xenografts from both direct orthotopic injection and serial transplantation lost the trimethylation at position 27 of histone H3, indicating that the models retained similar characters as the *H3F3A*-K27M mutant human DIPG (Figure 5).

## DISCUSSION

An optimal DIPG pre-clinical model should incorporate the following features: (1) a patient-derived origin retaining the characteristic of human DIPG; (2) a specific brainstem microenvironment created by precise orthotopic injection; (3) a cohort of resource which can be explicitly stratified in order to test novel therapeutic strategies; and (4) a stability and reproducibility which enables the model to be repeated according to a standard protocol. The objective of our study was approaching to these criteria as closely as possible.

In our study, several strategies were applied to assure the success of establishing cell lines resembled the characteristics of human DIPG. First, we chose treatment-naïve patients for a better preservation of original human DIPGs. Secondly, we used serum-free culture media with specified growth factors to avoid genetic alterations due to the presence of fetal bovine serum [29–31]. The eight DIPG cell lines we established following this protocol demonstrated various patterns in morphology, immunophenotype, gene mutation, proliferation capacity, and chromosome abnormality (Table 1). This result represented the heterogeneity of tumor origin in patients and provided a great source for understanding different molecular mechanisms of DIPG.

Development in cancer genomic sequencing greatly advanced the progression of molecular classification and targeted therapy of brain tumor. *H3F3A*, *TP53*, and *PPM1D* were the most frequently mutant genes in human DIPGs. Our established cell line maintained all these important genetic alterations from original tumors, which provided great opportunities for studying molecular mechanisms of DIPG. *H3F3A* was considered to be the most frequent and the most defining mutation associated with poor survival in DIPG [32], while *TP53*, *PPM1D*, *ACVR1* and *PIK3R1* were regarded as main driver mutations of DIPG as well [33]. The mutually exclusive presence of *TP53* and *PPM1D* mutations suggested distinct molecular pathways for tumorigenesis [27]. Our established eight DIPG cell lines can be stratified into three distinct subgroups by these three recurrent and significant mutation sites: (1) *H3F3A* mutation accompanied with *TP53* mutation (TT10603, TT10728, TT10902, TT11118, TT11201); (2) *H3F3A* mutation accompanied with *PPM1D* mutation (TT10630, TT10714); and (3) *H3F3A* mutation without *TP53* or *PPM1D* mutations (TT11111). This classification might provide clues on understanding clinical classification of DIPG at molecular level. Furthermore, given that both *H3F3A* and *PPM1D* were considered as therapeutic targets for DIPG, our DIPG cell lines not only can be served as great tools to study biological function of DIPG, but also can be used for novel drug screening. Interestingly, *TERT* promoter mutation at C250T was identified in patient TT10902 in both tumor and cell line, which may help understand the telomerase function in DIPG.

Glioma stem-like cells (GSLCs) display characteristics of self-renewal capacity and tumor-initiating ability [34, 35]. While several studies showed that serum condition may have a tendency of inducing differentiation and result in the loss of ability to generate diffuse infiltration in intracranial implantation [36, 37], the serum-free culture condition we used in this study may render it possible for DIPG cells to retain stem-like features. The immunohistochemistry we performed with multiple markers proved evidence of neural stem cell properties of these cells, representing the potential of differentiating to multiple lineages, while the large proportion of Ki67-positive cells demonstrated the capacity to proliferate. In addition, all eight cell lines could form neurospheres and be passaged in non-coated plates, indicating capacity of self-renewal. The fact that injection of as few as 10^3^ cells was sufficient to form intracranial tumors suggested these cells were highly tumorigenic. Altogether, the expression of stem cell markers, the ability of forming neurosphere and the capacity of forming tumors with a low cell number supported the conclusion that GSLCs existed in DIPG and may be the cause of DIPG's resistance to radiotherapy and chemotherapy.

The establishment of DIPG cell lines faithfully resembling the characteristics of human DIPG laid the foundation to the successful development of DIPG orthotopic xenograft models. Notably, these xenografts were generated by *in vitro* cultured cells instead of direct injection of fresh human tumor tissues specimens, owing to the result of an interesting study that direct injection of fresh specimens without *in vitro* culture will lead to murine but not human tumors [16]. MRI plays a significant role in the diagnosis of DIPG. The characteristic DIPG appearance on MRI is an infiltrative T2 high-signal lesion occupying more than half of the pons that often extends laterally into the cerebellar and vertically into the midbrain and medulla oblongata [38]. The established orthotopic xenograft models successfully recapitulated the imaging hallmarks of DIPG that resulted in infiltrated tumor gross within pons and invaded into adjacent areas. This result proved that MRI can be used as a non-invasive *in vivo* method to monitor the tumorigenesis progress in mice, same as it has been done in clinic for human patients. Together with the retaining of protein markers, the status of *H3F3A* mutation and the loss of trimethylation at position 27 of histone H3, these orthotopic models mimicked the anatomical, histological and molecular features of human DIPG.

The long latency of tumor formation by direct orthotopic injection could be a concern for functional and therapeutic research. Passaging as subcutaneous xenograft prior to orthotopic transplantation could maintain the signature of primary glioma cells while facilitating intracranial tumorigenesis[39–41]. In our study, serial transplantation not only preserved the characteristics of DIPG tumors, but also significantly decreased latency of tumorigenesis. One possible reason may be that the subcutaneous environment helped *in vitro* cultured cells adapt to *in vivo* condition before intracranial injection. Interestingly, tumors from serial transplantation also showed a more extensive infiltration pattern on MRI and higher level of neural stem cell markers, compared to that from direct orthotopic injection and original human specimens. Although the subcutaneous microenvironment was not identical to the CNS microenvironment and might result in overestimation of angiogenesis inhibitors [42], this serial transplantation model provided an efficient tool for future functional research and therapeutic exploration.

In summary, we successfully established sustainable patient-derived DIPG pre-clinical models, including cell lines as well as orthotopic xenografts through direct injection and serial transplantation. These reproducible models will not only help us make insights into the biological characteristic and molecular mechanism of DIPG, but also be valuable tools for *in vitro* drug screening and *in vivo* drug testing. Therefore, the establishment of our faithful DIPG cell lines and xenograft models may shed light on future investigations for more effective therapeutic strategies of this devastating disease.

## MATERIALS AND METHODS

### The establishment of DIPG cell lines

Tumor tissues were dissected from patients during surgery and collected in sterile Hibernation media (Supplementary Table 1) and transported on ice to the laboratory within two hours. Each tumor sample was transferred into one 100-mm culture dish and washed several times with ice-cold Hibernation media. Blood clots and gross necrosis were removed while washing the sample. The tissue was dissociated mechanically by chopping into small pieces with scissors. After primary mincing, they were collected into a 15-ml conical tube and washed twice with ice-cold Hibernation media. Next, the tissue was digested in DMEM with DNase I (250 U/ml) and collagenase type IV (1 mg/ml) (Invitrogen) for 30–60minutes at 37°C. The ratio of enzyme mixture solution and mechanically dissociated sample volume was 5:1. The primary tissue was triturated with intermittent pipetting every 10 minutes until there was no visible mass in the solution, to obtain single cell suspension. Cell suspension was centrifuged at 450g for 10 min at 4°C. Cells were washed twice with cold DMEM and were resuspended in Hibernation medium. Cells were then plated in PLO/Laminin coated 6-well plate. Serum-free medium composition was as following: DMEM (GIBCO, Invitrogen), L-glutamine, sodium pyruvate, B27, N2, insulin (20ug/ml), bFGF (20ng/ml), EGF (20ng/ml), PDGF-AB (20 ng/ml) and heparin (10 ng/ml)(Invitrogen). Cells were refed every three days and passaged every 1–2 weeks.

### Immunofluorescence staining

Coverslips, coated with PLO/Laminin (3ug/ml), were placed into 24-well plates and seeded with 4 × 10^4^ DIPG cells. After 48 hours, cells were fixed with 4% paraformaldehyde and blocked with 5% BSA, then incubated with primary antibody overnight at 4°C. After that, cells were rinsed with PBS and incubated with fluorescence-labeled secondary antibody (1:2000; Invitrogen) for 45min at room temperature. Meanwhile, cells were counterstained with DAPI. After rinsing with PBS, coverslips were mounted on slides with Fluoromount-G (SouthernBiotech). Images were obtained using a Zeiss microscope. Primary antibodies Ki67 (1:400; Thermo Scientific), Nestin (1:500; Millipore), GFAP (1:600; Sigma), Olig2 (1:500; Millipore) and PDGFRα (1:100; Santa Cruz) were used.

### Genotyping of cell lines and tumor tissues

Sanger sequencing of frequently mutant genes in DIPG were performed for all eight established DIPG cell lines and their corresponding human tumors. The gene list includes *H3F3A* (K27, G34), *HIST1H3B* (K27), *TP53* (all exons), *PPM1D* (exon 6), *TERT* promoter region (C228, C250), *ACVR1* (R206, R258, G328, G356), *PIK3CA* (E545, H1047), *PIK3R1* (N564) and *IDH1* (R132). Sanger sequencing of *H3F3A* (K27, G34) was performed for xenograft samples. The sequencing was performed by Genotron Health (Bejing) Co. Ltd, (Beijing, China).

### Growth curve and doubling time measurement

DIPG cells were seeded and cultivated in 96-well plates (5,000 cells /well) on day 0. Every other day, the plate was added 10μl CCK-8 per well and incubated for 4 hours in the incubator. Then the absorbance was measured at 450nm using a microplate reader. The growth curve was plotted using absorbance values and each point referred to triplicates. Cell doubling time was calculated by the following formula: doubling time=ln(2) · t/ln(N(t)/N(0)) t=10, where n was the cell number and t was the hours in culture.

### Karyotype analysis

DIPG cells were cultured to about 70% confluency, then treated with colchicine (25ug/ml) for 6h and preheated 0.075mM Kcl for 30 min at 37°C. After that, cells were added with fresh stationary liquid (methanol: glacial acetic acid=3:1) and centrifuged at 1200 rpm for 10 min. Then the pellets were fixed at room temperature for 10 min and 500ul stationary liquid was added to resuspend them. Cell droplets were applied to clean microslides that had been soaked in 4 to 6°C ice water. The samples were baked in 80°C constant temperature drying oven for 15 min. After Giemsa staining, the microslides were assessed by optical microscopy.

### The establishment of DIPG xenograft models

For brainstem injection, NGS mice were performed intraperitoneal injection of chloral hydrate for anesthesia and positioned into a digital stereotaxic frame (Stoelting). A 1.5cm midline skin incision was made over the posterior skull of the animal. Lambda was located as landmark. A small hole was created by puncture with an electric drill. A glass capillary loaded with 10^5^ cells in 5ul media was used to perform the injection under the control of Nanoject III Programmable Nanoliter Injector (Drummond Scientific Company). The coordinate was 1.5mm posterior to lamda, 1.0mm lateral to midline and 4.5mm beneath the skull. Syringe was slowly removed to prevent efflux. A small amount of bone wax was applied to the skull where the needle penetrated, and skin was sutured. After the injection, weight was recorded and mice were checked every 48 hours. Every two weeks or whenever any symptoms were identified, MRI was performed by 7.0-T mouse MRI. Mouse was sacrificed when tumor was confirmed by MRI or nine months after injection.

As for serial xenograft, 10^6^ DIPG cells were injected into the flank of NSG mice, and the tumor volume was measured with caliper every 2-3 days. Once tumor diameter reached 1.5cm, tumors were dissected and digested before being injected into the brainstem of NSG mice.

### Magnetic resonance scanning

MRI experiments were performed in a 7.0-T magnet (Bruker, Germany). A surface coil (2.3 cm × 1.5 cm) was used for brain imaging. Axial, coronal and sagittal T2-weighted images were acquired using a SE sequence (TE = 41 ms, TR = 3140 ms, FOV = 40 × 40 mm2, matrix size = 240 × 320, slice thickness = 0.7 mm, FA = 180°).

### Histopathology and immunohistochemistry staining

The tissues were fixed in 10% formalin, followed by dehydration in ethanol gradients, permeabilized with xylene and paraffin embedded. Sections with 5μm thickness were deparaffinized with xylene, hydrated with ethanol gradient and stained with hematoxylin and eosin (H&E) (MXB Biotechnologies). Immunohistochemical staining for Nestin (1:500; Millipore), Sox2 (1:500; Cell Signaling), Olig2 (1:500; Millipore), PDGFRα (1:100; Santa Cruz), GFAP (1:600; Sigma) and H3K27me3 (1:1000; Millipore) was performed. Endogenous peroxidase inactivation was carried out with 3% H_2_O_2_, and antigen retrieval was performed using heated sodium citrate treatment. Afterward, primary antibodies were added to each slide at appropriate dilutions, and the sections incubated with polyperoxidase-labeled secondary antibodies for 25 minutes. The final signals were developed using the 3, 3’-diaminobenzidine substrate (DAB) (MXB Biotechnologies). The sections were analyzed by optical microscopy after counterstaining with hematoxylin.

## SUPPLEMENTARY MATERIALS FIGURES AND TABLES


