# Supplementary material for: Patient-derived DIPG cells preserve stem-like characteristics and generate orthotopic tumors
Source: Oncotarget. 2017 Jul 28;8(44):76644–55. doi: 10.18632/oncotarget.19656 (PMC5652732; doi:10.18632/oncotarget.19656)
Supplement: Supplementary file 1 [file oncotarget-08-76644-s001.pdf]

Patient-derived DIPG cells preserve stem-like characteristics and generate orthotopic tumors

SUPPLEMENTARY MATERIALS

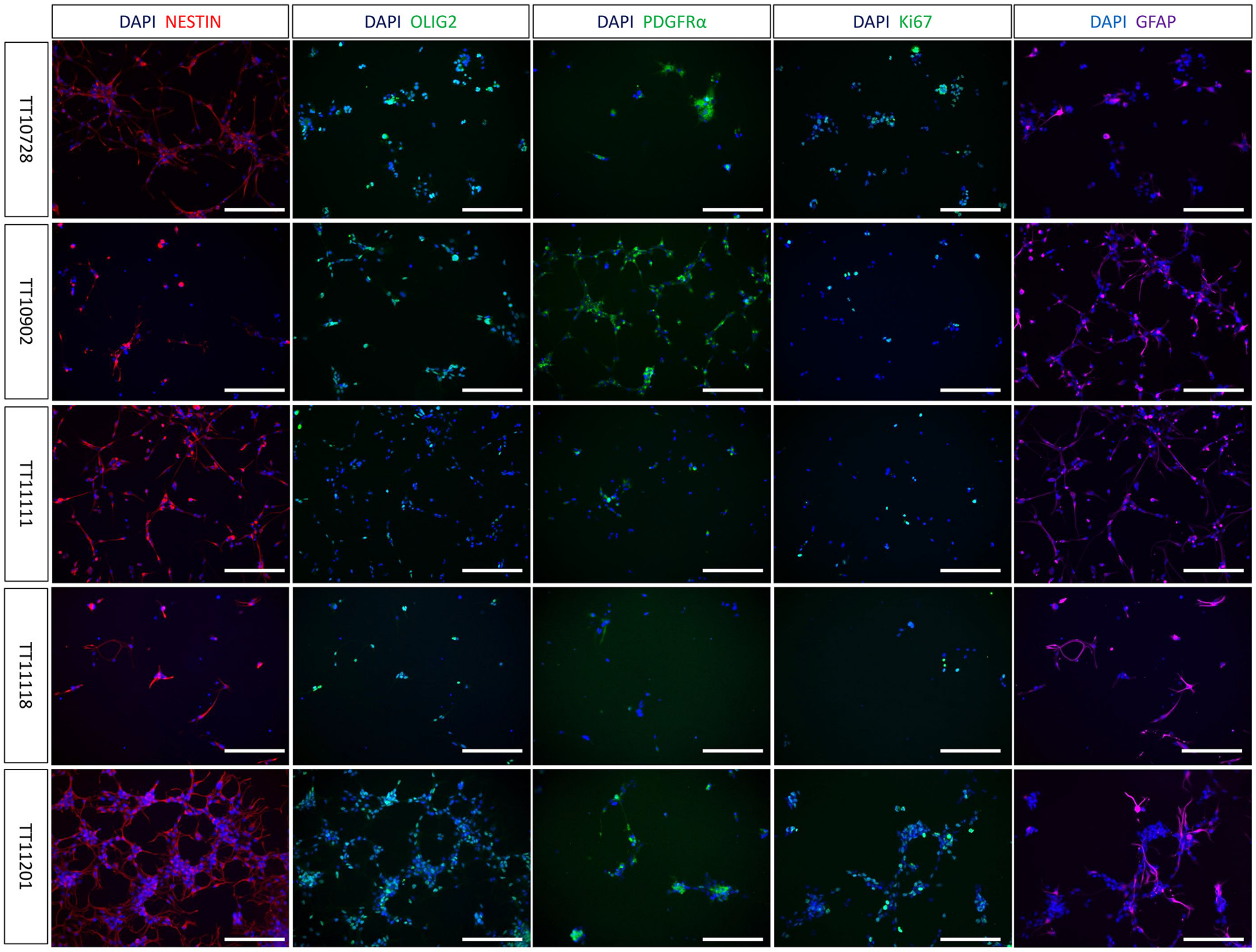

Supplementary Figure 1: Immunocytochemistry staining of DIPG cell lines TT10728, TT10902, TT11111, TT11118 and TT11201 (20X). (Scale bars: 200µm).

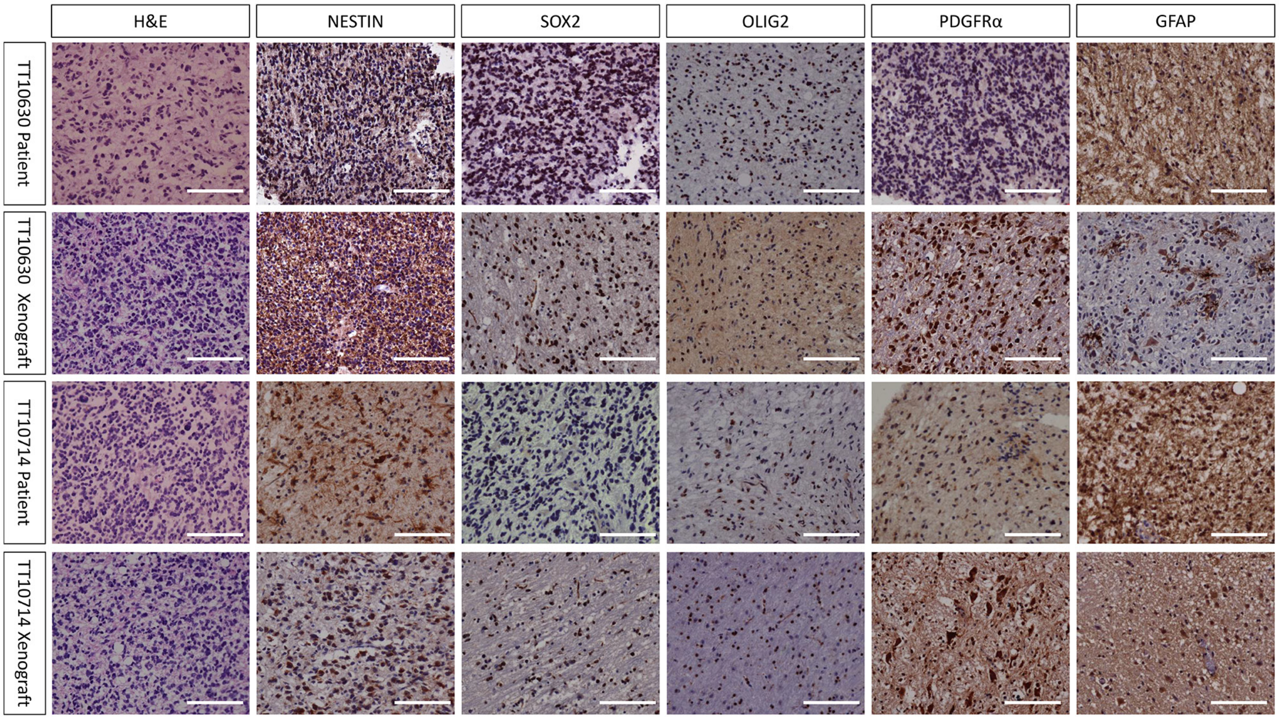

Supplementary Figure 2: Histopathological characteristic of TT10630 and TT10714 patient specimens and orthotopic xenografts (20X). (Scale bars: 200µm).

Supplementary Table 1: Hibernation medium

g/liter	mM	Chemical
2.24	30	KCl
0.2	5	NaOH
0.6	5	NaH_2PO_4 anhydrous
0.1	0.5	$MgCl_2$ X 6 H_2O
2.2	20	Na Pyruvate
1.0	5.5	Glucose
36.4	200	Sorbitol

pH to 7.3-7.4 with NaOH. Filter sterilize with 0.22 um filter.
